# Impact of Oral Clonidine on Duration of Opioid and Benzodiazepine Use in Mechanically Ventilated Children: A Randomized, Double-Blind, Placebo-Controlled Study

**DOI:** 10.22037/ijpr.2019.14862.12705

**Published:** 2019

**Authors:** Sara Salarian, Raha Khosravi, Ghamartaj Khanbabaei, Bahador Bagheri

**Affiliations:** a *Pediatric Pathology Research Center, Research Institute for Children Health, Mofid Children Hospital, Shahid Behehsti University of Medical Sciences, Tehran, Iran. *; b *Cancer Research Center, Semnan University of Medical Sciences, Semnan, Iran. *; c *Department of Pharmacology, School of Medicine, Semnan University of Medical Sciences, Semnan, Iran.*

**Keywords:** Clonidine, Sedation, Ventilation, Pediatric intensive care

## Abstract

Long term use of opioids and benzodiazepines are associated with important untoward effects. The α2 adrenergic agonist clonidine has sedative effects. Our goal was to study clonidine addition to total doses of fentanyl and midazolam and duration of ventilation in pediatric ICU (PICU). This randomized, double-blind, and placebo-controlled trial was conducted in PICU of Mofid Children Hospital. Hundred children aged from 2 to 15 years were randomized in 1:1 ratio to receive 5 μg/kg oral clonidine every 6 h or placebo plus 1-5 µg/kg/hr IV fentanyl and 0.05- 0.1 mg/kg/hr IV midazolam. Daily use of fentanyl and midazolam were measured. Ramsay sedation score was used for evaluation of sedation. A total of 96 patients were studied. The patients in placebo group received more midazolam and fentanyl compared with the patients in intervention group. Mean total dose of midazolam was 4.3 ± 2.2 mg in the placebo group and 2.7 ± 2.9 mg in the intervention group (*P*<0.05). Mean total dose of fentanyl was 34.4 ± 23.1 µg in the placebo group and 18.9 ± 10 µg in the intervention group (*P*<0.01). No significant differences were observed in duration of ventilation and length of PICU stay. No case of severe adverse effects was seen. This trial showed a reduction in total doses of midazolam and fentanyl given in ventilated children who were administered clonidine as add-on therapy. Clonidine addition had no effect on duration of mechanical ventilation.

## Introduction

Ventilation is a very important tool in critically ill children. Thirty to 64% of admitted infants and children may undergo mechanical ventilation in Pediatric Intensive Care Unit (PICU) (1). Adequate sedation is necessary for proper ventilation, and for prevention of unplanned-extubation. Unplanned-extubation is associated with hypoxia and ischemia and can damage the trachea and make the secondary extubation more difficult (1, 2). 

Current sedatives are problematic particularly for long-term use. Of note, excessive sedation is associated with pulmonary and cardiovascular depression, tolerance, and dependence (3). Clonidine, dexmedetomidine, fentanyl, midazolam, and propofol are current sedative drugs used in PICU (4). Prolonged ICU stay, higher incidence of delirium, higher incidence of withdrawal, and increased days on ventilator are negative outcomes of benzodiazepines and opioids (5). In general, benzodiazepines are the first line drugs for sedation due to acceptable sedative and hypnotic effects (2, 5). Benzodiazepines enhance γ-aminobutyric acid (GABA) induced ionic channels. Tolerance, withdrawal and respiratory depression are significant untoward effects of benzodiazepines like midazolam (5, 6). Fentanyl is a popular agent for analgesia in PICU. As with other opiates, tolerance, withdrawal, and respiratory depression can be seen in the patients receiving fentanyl (7, 8).

Clonidine, an imidazoline, activates α2 receptors in the lower brainstem region. Clonidine stimulates parasympathetic outflow and diminishes adrenergic drive, which can decrease the heart rate. Known as an antihypertensive drug, clonidine can cause sedation, anxiolysis, and analgesia. Clonidine carries an important side effect profile; hypotension and bradycardia (9). Addition of Clonidine to benzodiazepines and opiates may cause a reduction in the rate of unwanted effects like tolerance and withdrawal. Several studies have provided evidence for clonidine use in ICU and as add-on therapy in adults (10, 11). However, current evidence supporting efficacy and tolerability of clonidine in PICU remains scarce. This was the goal of our work. This study was implemented to investigate the impact of clonidine addition on the total dose of drugs to attain target sedation level in mechanically ventilated children according to the standard care of study center.

*Methods*

*Study design*

This randomized, double-blind, placebo-controlled, parallel-group, and single center study was conducted in PICU of Mofid Children Hospital, Tehran, Iran. Local Ethics Committee approved the study and written informed consent was obtained from parent (s) or by the legal representative prior to trial participation. The study was performed according to the World Medical Association Declaration of Helsinki and registered in Iranian Registry of Clinical Trials (IRCT20170920036296N1). 

The children aged from 2 to 15 years requiring mechanical ventilation were included. All participants and study personnel were masked to treatment allocation. Sealed envelopes with an enclosed assignment were used for allocation concealment. Randomization was done using a computer generated sequence list. Patients were randomly allocated in 1:1 to 5 μg/kg clonidine (Tolid Daru Pharmaceuticals, Iran) tablet dissolved in water every 6 h given via nasogastric tube (NGT) or identical placebo (Amin Daru, Iran) plus 1-5 µg/kg/hour fentanyl (Caspian Pharmaceuticals, Iran) and 0.05- 0.1 mg/kg/hour midazolam. Bolus doses of fentanyl and midazolam were used in cases with inadequate level of sedation. 

The participants were excluded if they had liver disorders (high LFT values), severe renal failure (high creatinine level according to age), receiving inotropes, severe neurological disorders (any prenatal anomalies), hemodynamic instability, AV block, receiving rescue therapies, inability to receive drugs through enteral route, adverse drug reactions, requiring less than 24 h sedation. The patients were discontinued from the study for these reasons: voluntary discontinuation, lost to follow-up, and safety.

According to our current protocol, the level of sedation was evaluated by Ramsey sedation score (12) and target sedation level was considered between 2 and 3. Evaluation of the sedation score was done every 1 hour and prior to any change in medication. The study medications were titrated to maintain the target sedation level. Sedation was continued for a maximum of 21 days. At the time of extubation, sedative medications were stopped. The study patients were followed until discharge from PICU.

*Efficacy assessment*

The primary end point was total doses of the drugs to attain target sedation level. The length of PICU stay from randomization until discharge and adverse effects of treatments were the secondary end points.

*Safety assessment*

Untoward effects and vital signs were monitored during patient’s stay in PICU. The participants were monitored for respiratory depression, hypotension, and bradycardia.

*Data analysis*

According to the assumption of 10% dropout in number of the patients with an α of 0.05 and β of 0.2, the total number of 110 patients was calculated for randomization. X^2^ test was used to study the differences between groups and repeated measures ANOVA was used for changes within the groups. Level of 0.05 was statistically significant. Analysis was carried out using SPSS software version 19.0, Chicago, USA.

## Results

*Baseline characteristics*

**Figure 1:**
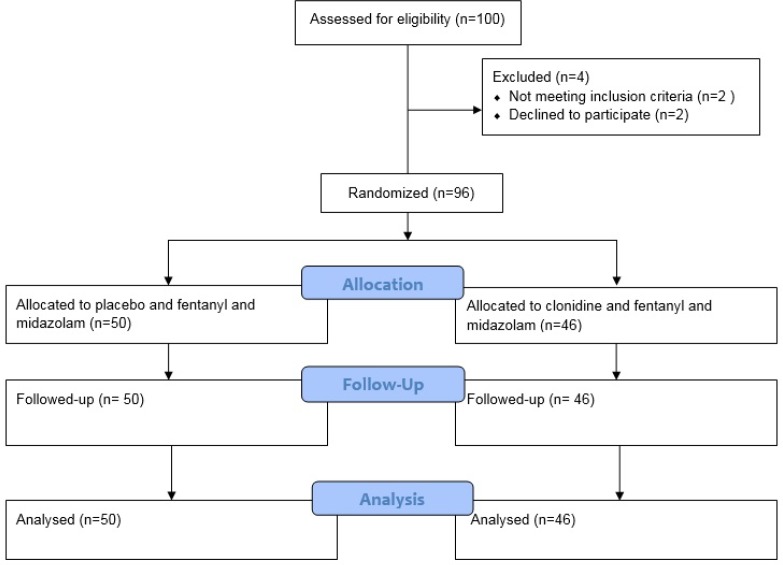
Consort diagram detailing study subjects

A total of 96 patients, out of 100 patients were recruited between April 2017 and September 2017. No children were lost to follow up and 96 patients were analyzed. Figure 1 shows consort chart of the study. Baseline clinical characteristics of the patients are presented in Table 1. As shown in Table 1, no significant differences were observed in the patients’ characteristics.

*Clonidine impact on sedative use*

All of the patients were given midazolam and fentanyl. In both groups, the target sedation level was reached. There were no significant differences in time to reach the target sedation in both groups. The patients in the placebo group received more midazolam and fentanyl compared with the intervention group (Table 2). Differences between mean total doses of midazolam and fentanyl were significant. The mean total dose of midazolam was 4.3 ± 2.2 mg in the placebo group and 2.7 ± 2.9 mg in the intervention group (*P *< 0.05). The mean total dose of fentanyl was 34.4 ± 23.1 µg in the placebo group and 18.9 ± 10 µg in the intervention group (*P *< 0.01).

*Clonidine impact on duration of ventilation*

As shown in Table 2, mean duration of ventilation was 7.1 ± 1.1 days in the placebo group and it was 6.3 ± 1.7 days in the intervention group. 

The difference between two groups failed to reach a statistical significance (*P *= 0.08). 

*Clonidine impact on length of PICU stay*

The mean length of stay in the PICU from randomization until patients’ discharge was not significantly different in the 2 groups (placebo, 198 ± 29 h, vs intervention, 210 ± 24 h; *P *= 0.07, Table 2).

*Adverse effects*

No case of severe hypotension or bradycardia was reported in the intervention group. In the placebo group, respiratory distress was the most common adverse effect reported in 3 (6%) patients. No patient died and no patient was withdrawn due to severe adverse effects.

**Table 1:** Characteristics of patients at baseline in two groups

**characteristics**	**placebo (n = 50)**	**intervention (n = 46)**	***P *** **value**
Age, y	12.4 ± 4.3	11.7 ± 2.5	0.5
Age (range)	2-14 yr	2 -15 yr	0.2
Female	28 (56)	29 (63)	0.8
Male	22 (44)	17 (37)	0.4
Body weight (Kg)	13 ± 3.8	4.9 ± 14	0.4
Admission diagnosis			
Pneumonia	12 (24)	10 (21.7)	0.2
Sepsis	7 (14)	8 (17.3)	0.4
Trauma	11 (22)	14 (30.4)	0.5
Surgery	4 (8)	1 (2.1)	0.7
Other	3 (6)	4 (8.6)	0.4
Hemoglobin, g/L	101 ± 4.6	103 ± 4.1	0.3
Total WBC count, 109/L	7.1 ± 3.24	6.9 ± 2.2	0.6
Platelets, 109/L	223 ± 113	243 ± 119	0.7
SBP (mmHg)	110 ±7.2	111 ±7.1	0.4
DBP (mmHg)	60 ± 5.9	63 ± 6.4	0.5
Respiratory rate (breaths/min)	25 ± 3.1	27± 4.8	1.0
Heat rate (beats/min)	111 ± 1.6	107 ± 1.9	0.3

Data are shown as mean ± SD or number (%).

**Table 2:** Details of clonidine effects on sedative total doses, duration of ventilation, and PICU stay

	**Placebo (n = 50)**	**Intervention (n = 46)**	***P *** **value**
Clonidine total dose (µg)	0	39.9 ± 14.9	0.4
Midazolam total dose (mg)	4.3 ± 2.2	2.7 ± 2.9	<0.05
Fentanyl total dose (µg)	34.4 ± 23.1	18.9 ± 10	<0.01
Duration of infusion (hr)	53 ± 4.9	56 ± 9.9	0.9
Ramsay score (baseline)	1.1± 0.2	1 ± 0.1	0.3
Ramsay score (during study)	2.6 ± 0.9	2.6 ± 1	0.7
Time at target sedation (%)	68	71	0.4
Duration of ventilation (day)	7.1 ± 1.1	6.3 ± 1.7	0.8
Length of PICU stay (hr)	198 ± 21	210 ± 19	0.7

Data are shown in number or mean ± SD.

## Discussion

In this randomized and double-blind trial, we provided evidence for the helpful effects of clonidine addition to fentanyl and midazolam to achieve an acceptable level of sedation in children who were mechanically ventilated. There was a trend to reduce the daily requirements of fentanyl and midazolam in the patients who were received clonidine. This reduction is likely beneficial if clonidine therapy can be continued as long as the patients are mechanically ventilated. A work from Lopez provided the first evidence for sedative effects of clonidine in children underwent ventilation (13). This was single arm study on 14 children and only cases with respiratory failure had been included; therefore, it is difficult to generalize clonidine effects in the children with other underlying diseases. 

We observed that duration of ventilation was similar in the placebo group and intervention group. Duffet’s pilot trial on 50 children provided necessary factors for feasibility and clinical utility of large clinical trials on clonidine in children (14). This trial reported no significant difference in level of sedation and duration of ventilation in the children received clonidine added to a benzodiazepine and morphine or added to placebo. It is expected that the reduction in the cumulative doses of sedatives would be associated with earlier extubation. Clonidine may favor earlier extubation and may cause better arousability. Long duration of ventilation is a risk factor for unplanned extubation in PICU (15). Previously, we showed that the rate of unplanned extubation was lower in the children receiving a combination of fentanyl and midazolam compared to the patients who were given either fentanyl or midazolam (16). We showed that clonidine addition to current PICU sedatives was linked with shorter ICU stay. Of note, the difference was not significant. Others have found that there was no significant difference in the rate of withdrawal and ventilation duration in 59 children aged from 1 month to 36 months who received either clonidine as adjunctive sedative or placebo added to morphine and benzodiazepines (17). At present, there is no large study and the present study has included more patients compared to the previously published investigations. It seems that the current data about clonidine as add-on therapy in PICU are limited and not conclusive.

Clonidine has an acceptable pharmacokinetics profile. It is well absorbed after oral administration and has bioavailability of 75-90%. One to 3 h after an oral dose, its peak concentration is achieved (18). Of note, it has considerable analgesic effects. Dry mouth, and hypotension are two common side effects of clonidine. In particular, hypotension is more common in epidural administration of clonidine. AV block can be considered as the most dangerous untoward effect of clonidine therapy (19-21). In the current study, we observed no significant adverse effect like hypotension or bradycardia. 

A very important difference exists in cost of clonidine and other α2 agonists like dexmedetomidine which has gained a prominent role in ICU (22). Dexmedetomidine has much higher cost than clonidine. 

As we observed, the add-on therapy with clonidine had acceptable response with low rate of adverse effects. Of note, 3 patients in placebo group experienced a respiratory distress which may be due to larger doses of sedatives. More studies are needed to gain brighter understanding about sedative and analgesic effects of clonidine in pediatrics either as an adjunct drug or choice drug. In addition, efficacy and safety of higher doses of clonidine should be studied in future works. Our major strengths were wide ranges of age and admission reasons, double blinding, and frequent evaluation of sedation and untoward effects. Compared to the published works, larger population was included in our study.

*Study limitation*

The present study has a number of limitations. Due to limited number of patients, future studies should be dealt with sedative effects of clonidine in a bigger population providing a wide range of patients with different underlying disorders. We did not study delirium, arousability, and pain. They can be measured and investigated by future works.

## Conclusion

This trial showed a reduction in total doses of sedatives given in ventilated children who were on clonidine as add-on therapy. Clonidine addition had no effect on duration of mechanical ventilation. Due to low cost and availability, clonidine can be considered as an adjunct to the sedatives in PICU.
